# Supplementary material for: Impact of the COVID-19 Vaccination Program on case incidence, emergency department visits, and hospital admissions among children aged 5–17 Years during the Delta and Omicron Periods—United States, December 2020 to April 2022
Source: PLoS One. 2022 Dec 9;17(12):e0276409. doi: 10.1371/journal.pone.0276409 (PMC9733849; doi:10.1371/journal.pone.0276409)
Supplement: S1 Table — (DOCX) [file pone.0276409.s001.docx]

|  |  |
| --- | --- |

**Table S1.** Number and rates^a^ of COVID-19 cases, emergency department (ED) visits, and hospital admissions in the United States during the Delta and Omicron periods, by age group and overall (5 – 17 years).

| **Outcome** | **Delta** | | | | | | | | **Omicron** | | | | | | | |
| --- | --- | --- | --- | --- | --- | --- | --- | --- | --- | --- | --- | --- | --- | --- | --- | --- |
|  | **5 - 11 years** | | **12 - 15 years** | | **16 - 17 years** | | **All Pediatric Ages*** | | **5 - 11 years** | | **12 - 15 years** | | **16 - 17 years** | | **All Pediatric Ages^b^** | |
|  | **Count** | **Rate or Percent (ED)** | **Count** | **Rate** | **Count** | **Rate** | **Count** | **Rate** | **Count** | **Rate** | **Count** | **Rate** | **Count** | **Rate** | **Count** | **Rate** |
| Cases^c^ | 791,650 | 4,032 | 496,498 | 4,298 | 264,645 | 4,615 | 1,552,793 | 4,206 | 1,617,533 | 8,238 | 1,048,572 | 9,077 | 554,927 | 9,676 | 3,221,032 | 8,724 |
| ED Visits^d^ | 48,201 | 2.74% | 40,824 | 3.64% | 27,393 | 3.88% | 116,418 | 3.24% | 60,777 | 3.67% | 38,728 | 3.83% | 25,599 | 4.19% | 125,104 | 3.81% |
| Hospital Admission^e^ |  | | | | | | 27,551 | 37 |  | | | | | | 48,988 | 67 |

^a^ Rates for case and hospital admission data sources defined as number of cases or hospital admissions per 100,000 persons during time period of interest. Rate for ED data source defined as percentage of emergency department encounters with diagnosed COVID-19

^b^ Hospital admission data are only available in aggregate for children aged 0–17 years during these periods. For cases and ED visits, data are shown for pediatric patients aged 5–17 years.

^c^ Jurisdictions excluded from case data source include Alaska, Connecticut, District of Columbia, Florida, Kentucky, Hawaii, Iowa, Maryland, Missouri, Mississippi, Nebraska, New Hampshire, Oklahoma, Rhode Island, South Dakota, Texas, Wisconsin, and West Virginia

^d^ Jurisdictions excluded from emergency department data source include California, Hawaii, Iowa, Minnesota, Oklahoma, Missouri, and Maryland

^e^ No jurisdictions were excluded from hospital admission data source
