# Supplementary material for: Impact of the COVID-19 Vaccination Program on case incidence, emergency department visits, and hospital admissions among children aged 5–17 Years during the Delta and Omicron Periods—United States, December 2020 to April 2022
Source: PLoS One. 2022 Dec 9;17(12):e0276409. doi: 10.1371/journal.pone.0276409 (PMC9733849; doi:10.1371/journal.pone.0276409)
Supplement: S2 Table — (DOCX) [file pone.0276409.s002.docx]

**Table S2.** Demographic characteristics and county classification breakdowns for jurisdictions included vs. excluded for COVID-19 cases

| **Demographic Variable** | **Included Jurisdictions** | | | **Excluded Jurisdictions** | | |
| --- | --- | --- | --- | --- | --- | --- |
| **Age Group** | **N** | **Total** | **Percent** | **N** | **Total** | **Percent** |
| 5–11 | 19,635,272 | 36,922,385 | 53.18% | 8,810,824 | 16,540,082 | 53.27% |
| 12–15 | 11,552,181 |  | 31.29% | 5,171,345 |  | 31.27% |
| 16–17 | 5,734,934 |  | 15.53% | 2,557,913 |  | 15.46% |
| **Sex** | **N** | **Total** | **Percent** | **N** | **Total** | **Percent** |
| Female | 18,068,704 | 36,922,385 | 48.94% | 8,094,302 | 16,540,082 | 48.94% |
| Male | 18,853,681 |  | 51.06% | 8,445,780 |  | 51.06% |
| **CDC County Social Vulnerability Index (SVI)** | **N (Counties)** | **Total (Counties)** | **Percent** | **N (Counties)** | **Total (Counties)** | **Percent** |
| Highest Vulnerability Counties | 473 | 1,991 | 23.76% | 312 | 1,150 | 27.13% |
| Second Highest Vulnerability Counties | 502 |  | 25.21% | 283 |  | 24.61% |
| Second Lowest Vulnerability Counties | 524 |  | 26.32% | 261 |  | 22.70% |
| Lowest Vulnerability Counties | 492 |  | 24.71% | 294 |  | 25.57% |
| **NCHS Urban/Rural County Classification** | **N (Counties)** | **Total (Counties)** | **Percent** | **N (Counties)** | **Total (Counties)** | **Percent** |
| Large Central Metro | 48 | 1,991 | 2.41% | 20 | 1,150 | 1.74% |
| Large Fringe Metro | 269 |  | 13.51% | 99 |  | 8.61% |
| Medium Metro | 247 |  | 12.41% | 125 |  | 10.87% |
| Small Metro | 250 |  | 12.56% | 108 |  | 9.39% |
| Micropolitan | 410 |  | 20.59% | 230 |  | 20.00% |
| Noncore | 767 |  | 38.52% | 568 |  | 49.39% |
